# *QuickStats:* Percentage* of Adults Who Did Not Get Needed Dental Care Because of Cost in the Past 12 Months,^†^ by Age Group and Sex — National Health Interview Survey, United States, 2019^§^

**DOI:** 10.15585/mmwr.mm7025a5

**Published:** 2021-06-25

**Figure:**
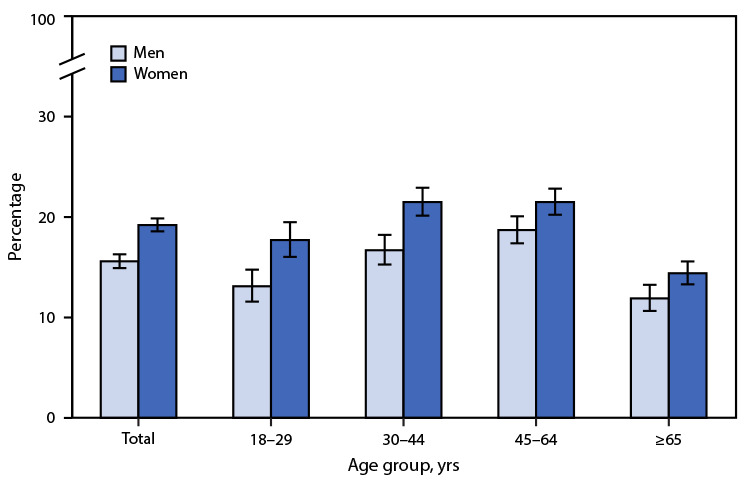
In 2019, among adults aged ≥18 years, women (19.2%) were more likely than men (15.6%) not to get needed dental care because of cost in the past 12 months. The difference by sex was seen for all age groups: 17.7% versus 13.1% among adults aged 1829 years, 21.5% versus 16.7% among those aged 30–44 years, 21.5% versus 18.7% among those aged 45–64 years, and 14.4% versus 11.9% among those aged ≥65 years. For both men and women, the percentages were highest among those aged 30–44 and 45–64 years. For men, the percentages were lowest among those aged 18–29 years and ≥65 years; for women, the percentage was lowest among those aged ≥65 years.

**Source:** National Center for Health Statistics; National Health Interview Survey, 2019. https://www.cdc.gov/nchs/nhis.htm

